# Supplementary material for: Production of Fungal Quinones: Problems and Prospects
Source: Biomolecules. 2022 Jul 28;12(8):1041. doi: 10.3390/biom12081041 (PMC9405642; doi:10.3390/biom12081041)
Supplement: Supplementary file 1 [file biomolecules-12-01041-s001.zip › biomolecules-1812990-supplementary.pdf]

Supplementary materials

Title: Production of fungal quinones - problems and prospects

Authors:

Johan Vormsborg Christiansen, Thomas Ostenfeld Larsen and Jens Christian Frisvad

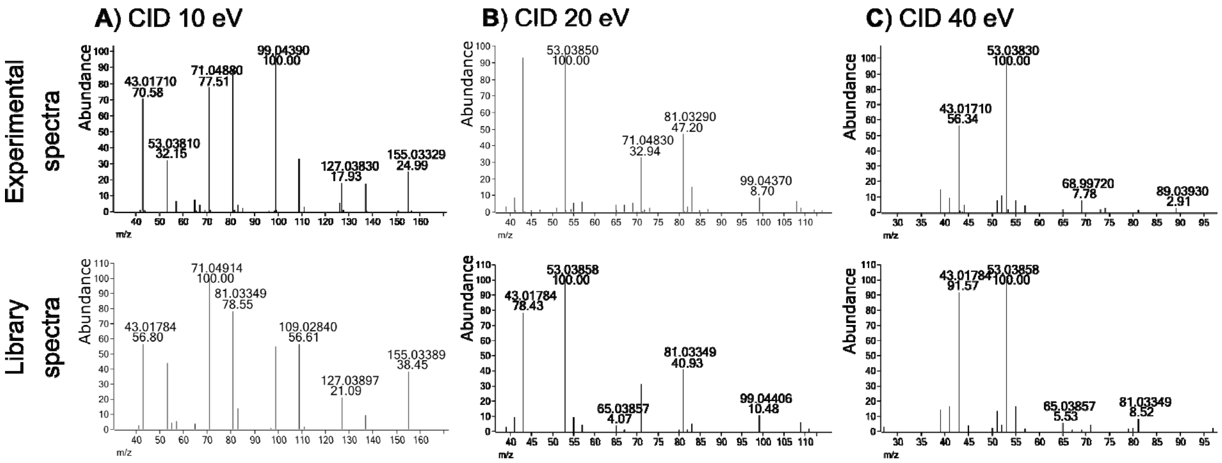

Figure S1. Comparison of tandem mass spectrometry (MS/MS) spectral data of patulin with an in-house MS/MS spectral library across three collision induced dissociation (CID) energies: 10, 20 and 40 eV, respectively (**A-C**). Top panels show the experimental spectra, while the bottom panels show the library spectra.

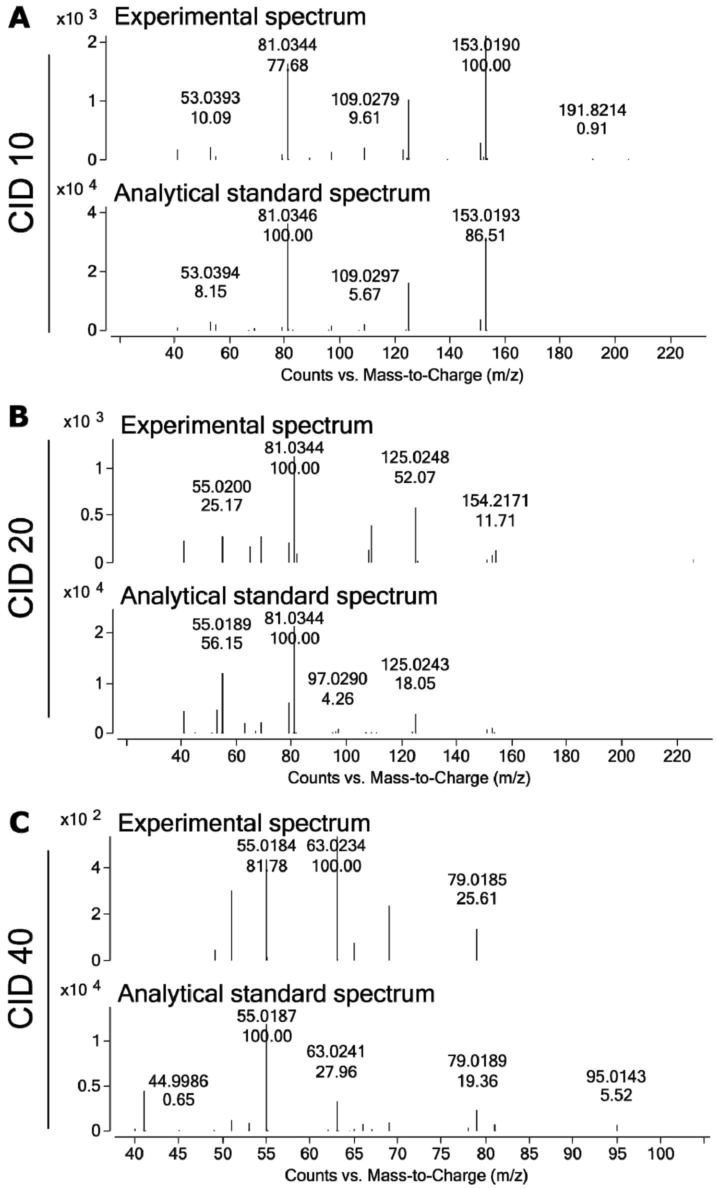

Figure S2. MS/MS spectral comparison of terreic acid (TA) at 10, 20 and 40 eV, respectively (**A-C**). Top spectra are the experimental spectra, while bottom spectra are from the TA analytical standard.
